# Exploring factors influencing patient mortality and loss to follow-up in two paediatric hospital wards in Zamfara, North-West Nigeria, 2016–2018

**DOI:** 10.1371/journal.pone.0262073

**Published:** 2021-12-31

**Authors:** Anna Maisa, Abdulhakeem Mohammed Lawal, Tarikul Islam, Chijioke Nwankwo, Bukola Oluyide, Adolphe Fotso, Harriet Roggeveen, Saskia van der Kam, Cono Ariti, Karla Bil, Annick Lenglet

**Affiliations:** 1 Médecins Sans Frontières (MSF), Sokoto, Nigeria; 2 MSF, Anka, Nigeria; 3 Anka General Hospital, Anka, Nigeria; 4 MSF, Abuja, Nigeria; 5 MSF, Operational Centre Amsterdam, Amsterdam, Netherlands; 6 Cardiff University, School of Medicine, Cardiff, United Kingdom; University of Washington, UNITED STATES

## Abstract

**Introduction:**

Child mortality has been linked to infectious diseases, malnutrition and lack of access to essential health services. We investigated possible predictors for death and patients lost to follow up (LTFU) for paediatric patients at the inpatient department (IPD) and inpatient therapeutic feeding centre (ITFC) of the Anka General Hospital (AGH), Zamfara State, Nigeria, to inform best practices at the hospital.

**Methods:**

We conducted a retrospective cohort review study using routinely collected data of all patient admissions to the IPD and ITFC with known hospital exit status between 2016 and 2018. Unadjusted and adjusted rate ratios (aRR) and respective 95% confidence intervals (95% CI) were calculated using Poisson regression to estimate the association between the exposure variables and mortality as well as LTFU.

**Results:**

The mortality rate in IPD was 22% lower in 2018 compared to 2016 (aRR 0.78; 95% CI 0.66–0.93) and 70% lower for patients coming from lead-affected villages compared to patients from other villages (aRR 0.30; 95% CI 0.19–0.48). The mortality rate for ITFC patients was 41% higher during rainy season (aRR 1.41; 95% CI 1.2–1.6). LTFU rates in ITFC increased in 2017 and 2018 when compared to 2016 (aRR 1.6; 95% CI 1.2–2.0 and aRR 1.4; 95% CI 1.1–1.8) and patients in ITFC had 2.5 times higher LTFU rates when coming from a lead-affected village.

**Conclusions:**

Our data contributes clearer understanding of the situation in the paediatric wards in AGH in Nigeria, but identifying specific predictors for the multifaceted nature of mortality and LTFU is challenging. Mortality in paediatric patients in IPD of AGH improved during the study period, which is likely linked to better awareness of the hospital, but still remains high. Access to healthcare due to seasonal restrictions contributes to mortalities due to late presentation. Increased awareness of and easier access to healthcare, such as for patients living in lead-affected villages, which are still benefiting from an MSF lead poisoning intervention, decreases mortalities, but increases LTFU. We recommend targeted case audits and qualitative studies to better understand the role of health-seeking behaviour, and social and traditional factors in the use of formal healthcare in this part of Nigeria and potentially similar settings in other countries.

## Introduction

Global under-five mortality has reduced from 91 deaths per 1000 live births in 1990 to 43 deaths per 1000 live births in 2015 [1]. The burden of under-five mortality disproportionately affects low- and middle-income countries, with half of the world’s total under-five deaths occurring in sub-Saharan Africa alone [2]. Paediatric inpatient mortality ranges from 3% to 21% across different Sub-Saharan countries and different hospital settings including specialised paediatric care [3–7]. Although Nigeria has seen a consistent reduction in mortality for children under five years of age from 213 per 1000 live births in 1990 to 104 per 1000 live births in 2015, childhood mortality remains high, with the national average reported to be around 120 per 1000 live births in 2017 [8–10].

Child mortality has been linked to infectious diseases, malnutrition and lack of access to essential health services [11, 12]. Chronic malnutrition in particular, evidenced by stunting and wasting, has been implicated in increased morbidity and mortality from infection in low resource countries [13, 14]. Malnutrition has also been found to be a predictor for mortality among hospitalised children under five years of age in Nigeria [15].

Patient discharge and death are not the only outcomes to be considered in low resource countries, as lost to follow-up (LTFU), which is defined as a patient leaving the hospital before being formally discharged, is also a non-negligible outcome. Some countries have reported LTFU rates between 4.4% and 16.6%, especially among children being treated for severe acute malnutrition [6, 16–18]. The factors associated with paediatric patients LTFU are less well described in the literature. The majority of studies around paediatric patient LTFU have been conducted in HIV infected children and have shown that this very specific patient population tends to be LTFU from hospital when they are younger than 2 years of age, undernourished and presenting late with advanced disease [19–21].

**Fig 1:**
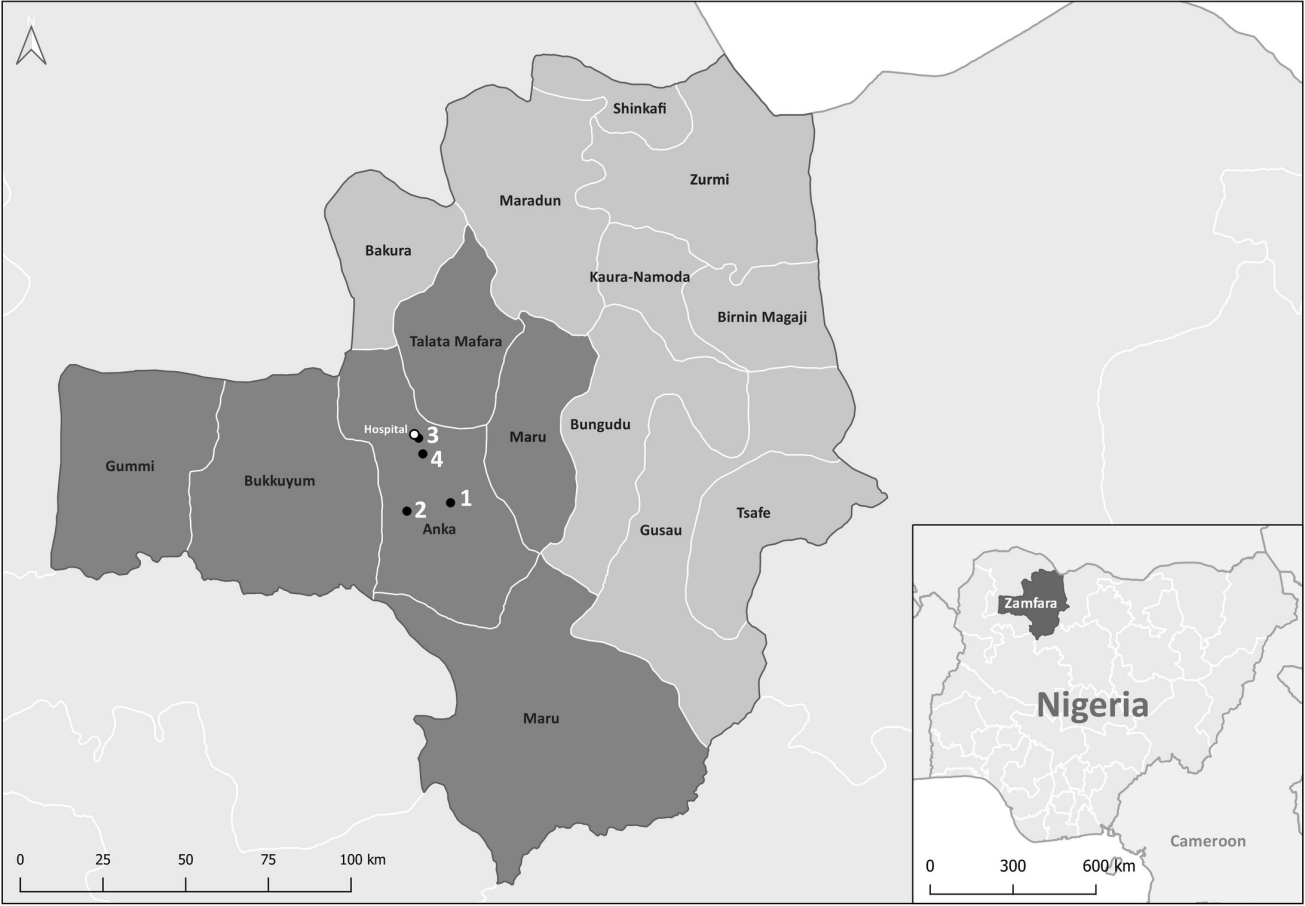
Nigeria map with Zamfara enlargement. Geolocation of origin of admitted patients to IPD and ITFC paediatric wards of the AGH (white dot), 2016–2018. Lead-affected villages (1–4) are shown as black dots. Five out of 14 LGAs (Anka, Bukkuyum, Gummi, Maru and Talata Mafara) in Zamfara State are shown in darker shading and with their ward borders. Map was created with QGIS and contains information from OpenStreetMap and OpenStreetMap Foundation, which is made available under the Open Database License.

Zamfara State in North-West Nigeria (Fig 1) has been experiencing civil unrest resulting in mass population displacement since 2014. Zamfara’s under-five mortality rate accounts for 210 deaths per 1000 live births and is therefore almost twice as high as the national average in Nigeria (120 deaths per 1000 live births) [8, 22]. Médecins Sans Frontières (MSF) has been working in collaboration with the Ministry of Health (MOH) in Anka Local Government Area (LGA) in Zamfara State since 2010 following a lead poisoning outbreak [23, 24]. MSF supports multiple health-related activities in the villages in Anka LGA that were affected. These include lead poisoning treatment and monitoring, primary healthcare and an Ambulatory Therapeutic Feeding Centre (ATFC). Since 2014, MSF also collaborates with the MOH in the inpatient paediatric department (IPD), the inpatient therapeutic feeding centre (ITFC) and the paediatric isolation ward at Anka General Hospital (AGH), the secondary healthcare facility for the LGA.

We aimed to explore the clinical profile of paediatric patients, who died or were LTFU following an admission to AGH. In addition, we attempted to identify predictors for these outcomes in hospitalised children. We hope that a better understanding of these negative outcomes in paediatric patients will contribute to improved patient care at AGH and potentially help to educate other facilities in Nigeria or other countries facing similar challenges.

## Methods

### Study design

We conducted a retrospective cohort review study using routinely collected data. All patient admissions to the IPD and ITFC paediatric wards of the AGH with a known hospital exit status from 1 January 2016 to 31 December 2018 were included. The data was accessed during January until September 2019. The data for December 2016 was excluded from our analysis due to missing mortality data in that month. We assume that this was a data encoding error, rather than no deaths occurring during that month.

The paediatric department of AGH is made up of three wards: the inpatient department (IPD), which includes the isolation ward, and the inpatient therapeutic feeding centre (ITFC). At the time of the study period, the number of health care workers (including medical doctors, nurses, nutrition and clinical assistants, laboratory technicians and hygiene agents) working in the different wards increased from 46 in 2016 to 92 in 2018 during dry season and from 75 in 2016 to 119 in 2018 during rainy season. There were 90–95 beds available, up to 47 in ITFC, 32 in IPD and 16 in isolation.

The data of the study is based on routinely collected clinical data of the Anka hospital for paediatric patient admissions. All data was entered in a registration book and in the medical records for the patient, as well as into a digital health information system. All exported data from the health information system was anonymised and password protected before analysis, and was only accessible for the study team. The data used for analysis included patient identification number (for deduplication), age, sex, village of residence, admission date, exit date, ward, primary diagnosis, exit code, length of hospital stay, time to death, and referral information. Village of residence included lead-affected villages, which are still benefiting from the MSF lead-poisoning intervention, potentially leading to higher awareness of healthcare. Duplicate entries of a combination of patient identification number, admission date and sex were excluded.

The exit code (i.e., clinical outcome) for patients leaving the hospital were categorised as death, LTFU, referral or discharge. LTFU was defined as a patient leaving the hospital before being formally discharged. This included patients, for whom we were missing information on the clinical outcome, patients, whose caregivers discharged them against medical advice and those that defaulted from care. We considered LTFU when there was no exit code available for a particular patient.

Patients admitted to the IPD and ITFC were analysed separately due to the differences in admission criteria for these two wards.

### Statistical analysis

Characteristics of admitted patients were described for each time period, age group, sex, diagnosis (S1 Table) and patient origin stratified by ward. We calculated the proportion of deaths and LTFU for each patient characteristic. Differences between the proportions of outcomes in both hospital wards were assessed using Pearson’s chi-squared test.

The outcome variables were death and LTFU. For each admission we calculated person-time as the days from date of admission until date of death or LTFU. For death, patients were censored at time of LTFU and for LTFU at date of death. To allow for the changing risk of death from time since admission the person-days of follow-up were split into the following time intervals: less than 24h, 24h and less than 48h, 48h and less than 72h, 72h and less than 96h, and 96h or more. Mortality rates and 95% confidence intervals were calculated for each time period, age group, sex, diagnosis and patient origin for each ward.

Time to event analysis was used to describe mortality and LTFU rates in the 3-year study period. Crude and adjusted rate ratios (RR) were calculated using Poisson regression models. The assessment of the risk of death from time since admission was calculated using a Poisson model parametrised as a piecewise exponential model. All variables with p<0.2 in the univariate analysis were included in the multivariable model and eliminated in a backward stepwise fashion using a likelihood-ratio test at each step until all remaining factors were significant at a 0.05 level. Excluded variables were also checked for inclusion.

All possible interactions between covariates were assessed using the likelihood-ratio test and consideration of the relevance and plausibility of the interaction. However, no plausible interactions emerged from the analysis.

Data was cleaned and analysed using Microsoft Corporation 2016, Microsoft Excel (Retrieved from https://office.microsoft.com/excel)and StataCorp. 2017, Stata Statistical Software: Release 15, College Station, TX: StataCorp LLC.

#### Ethics approval and consent to participate

This research fulfilled the exemption criteria set by the Médecins Sans Frontières Ethics Review Board for a posteriori analyses of routinely collected clinical data and thus did not require MSF Ethics Review Board review. It was conducted with permission from Sidney Wong, Medical Director, Operational Centre Amsterdam (OCA), Médecins Sans Frontières.

## Results

### Patient characteristics

Between 1 January 2016 and 31 December 2018, 13654 (56%) patients exited the IPD and 10744 (44%) exited the ITFC ward (Table 1). Comparison of the patients of IPD and ITFC showed significant differences in characteristics for each outcome (S2 Table). The proportion of both deceased and patients LTFU was higher in ITFC (9.4% and 3.7%) compared to IPD (6.5% and 2.7%). As a result of these differences, patient deaths and LTFUs from these two wards were analysed separately.

**Table 1:** Demographic characteristics and proportion of patient deaths and LTFUs in IPD and ITFC.

		Total	IPD						ITFC					
		admissions	admissions		Deaths		LTFU		admissions		Deaths		LTFU	
		n	n	%	n	%	N	%	n	%	n	%	n	%
**Overall**		24398	13654	55.96	887	6.50	373	2.73	10744	44.04	1010	9.40	399	3.71
**Age**	0–6 months	2261	1836	81.20	174	9.48	76	4.14	425	18.80	35	8.24	19	4.47
	7–12 months	6931	3503	50.54	201	5.74	107	3.05	3428	49.46	259	7.56	124	3.62
	13–24 months	9959	4430	44.48	228	5.15	117	2.64	5529	55.52	545	9.86	208	3.76
	25–36 months	3096	1982	64.02	140	7.06	34	1.72	1114	35.98	150	13.46	42	3.77
	37–48 months	985	856	86.90	68	7.94	20	2.34	129	13.10	11	8.53	5	3.88
	49–60 months	606	546	90.10	40	7.33	5	0.92	60	9.90	5	8.33	0	0
	5+ years	560	501	89.46	36	7.19	14	2.79	59	10.54	5	8.47	1	1.69
**Sex**	Male	13201	7512	56.90	487	6.48	216	2.88	5689	43.10	502	8.82	213	3.74
** **	Female	11197	6142	54.85	400	6.51	157	2.56	5055	45.15	508	10.05	186	3.68
**Year of Exit**	2016	6135	3328	54.25	212	6.37	84	2.52	2807	45.75	248	8.84	83	2.96
	2017	8840	5195	58.77	308	5.93	162	3.12	3645	41.23	336	9.22	139	3.81
	2018	9423	5131	54.45	367	7.15	127	2.48	4292	45.55	426	9.93	177	4.12
**Month of**	January	1351	858	63.51	45	5.24	17	1.98	493	36.49	26	5.27	19	3.85
**Exit**	February	1545	979	63.37	69	7.05	50	5.11	566	36.63	53	9.36	19	3.36
	March	1935	1131	58.45	73	6.45	32	2.83	804	41.55	66	8.21	17	2.11
	April	2015	1088	54.00	77	7.08	43	3.95	927	46.00	85	9.17	45	4.85
	May	2026	1078	53.21	76	7.05	31	2.88	948	46.79	81	8.54	44	4.64
	June	1788	790	44.18	43	5.44	16	2.03	998	55.82	106	10.62	47	4.71
	July	2404	1285	53.45	87	6.77	33	2.57	1119	46.55	136	12.15	35	3.13
	August	2353	1185	50.36	77	6.50	33	2.78	1168	49.64	142	12.16	46	3.94
	September	2482	1357	54.67	91	6.71	33	2.43	1125	45.33	112	9.96	40	3.56
	October	2897	1689	58.30	119	7.05	45	2.66	1208	41.70	97	8.03	35	2.90
	November	2416	1457	60.31	81	5.56	30	2.06	959	39.69	66	6.88	33	3.44
	December	1186	757	63.83	49	6.47	10	1.32	429	36.17	40	9.32	19	4.43
**Season**	Dry season	17159	9827	57.27	632	6.43	274	2.79	7332	42.73	620	8.46	278	3.79
	Rainy season	7239	3827	52.87	255	6.66	99	2.59	3412	47.13	390	11.43	121	3.55
**Number of**	0 days	564	384	68.09	235	61.20	34	8.85	180	31.91	124	68.89	19	10.56
**days**	1 day	1451	1055	72.71	246	23.32	49	4.64	396	27.29	201	50.76	34	8.59
**hospitalized**	2 days	4358	3642	83.57	154	4.23	36	0.99	716	16.43	145	20.25	29	4.05
	3 days	4251	2847	66.97	87	3.06	54	1.90	1404	33.03	122	8.69	48	3.42
	4 days	3891	1992	51.20	42	2.11	59	2.96	1899	48.80	77	4.05	38	2.00
	5 days	2877	1220	42.41	32	2.62	31	2.54	1657	57.59	65	3.92	26	1.57
	6 days	2115	823	38.91	26	3.16	14	1.70	1292	61.09	61	4.72	57	4.41
	7 days	1465	574	39.18	11	1.92	26	4.53	891	60.82	59	6.62	32	3.59
	8+ days	3426	1117	32.60	54	4.83	70	6.27	2309	67.40	156	6.76	116	5.02
**Primary**	Malaria	7695	7695	n/a	465	6.04	124	1.61	n/a					
**diagnosis** ^**a**^	Sepsis	629	629		99	15.74	24	3.82						
** **	LRTI	896	896		80	8.93	22	2.46						
** **	Meningitis	203	203		45	22.17	22	10.84						
** **	Measles	1725	1725		38	2.20	16	0.93						
** **	Neonatal Disease	272	272		37	13.60	15	5.51						
** **	Gastroenteritis	979	979		27	2.76	11	1.12						
** **	Tetanus	60	60		18	30.00	7	11.67						
** **	Liver disease	21	21		12	57.14	6	28.57						
** **	Anaemia	55	55		10	18.18	4	7.27						
	Other	847	847		52	6.14	37	4.37						
**Patient origin** ^**b**^	Lead-affected village	1291	1077	83.42	19	1.76	48	4.46	214	16.58	15	7.01	16	7.48
	Other villages	23105	12577	54.43	868	6.90	325	2.58	10528	45.57	994	9.44	383	3.64

n/a–not applicable; LRTI–lower respiratory tract infection.

^a^ information missing on 316 of 24398 patients.

^b^ information missing on 2 of 24398 patients.

The median age of patients was 18 months in both wards. The male to female ratio was 1.2 in IPD and 1.1 in ITFC.

Patient admissions increased each year in ITFC compared to IPD, which showed similar admission numbers in 2017 and 2018. Overall, more patients were admitted to IPD than to ITFC (Table 1).

Most patients were admitted during the months of the rainy season (July, August and September) and there was a higher proportion of deaths during the rainy season compared to the dry season in both wards, especially in ITFC (11.4% vs 8.5%, p<0.0001) compared to IPD (6.7% vs 6.4%, p = 6.2) (Table 1; S2 Table).

The median length of stay in both hospital wards was 4 days (IQR 2–6). The median length of stay was shorter in IPD than in ITFC (3 days vs 5 days, p<0.0001).

The ITFC only admitted children who had a diagnosis of acute malnutrition. The top 5 primary diagnoses in IPD were malaria (56%), followed by measles (13%), gastroenteritis (7%), lower respiratory tract infections (LRTI; 7%) and sepsis (5%).

The geolocation of origin of the patients seen at the Anka IPD and ITFC wards, comprises an area covering five different Local Government Areas (LGAs; Fig 1) in Zamfara state. This area includes the four lead-affected villages (Fig 1, points 1–4), which are still benefiting from MSF outreach activities.

### Mortality rates

The crude mortality rates were calculated for each ward and patient characteristic (Table 2). The mortality rates in IPD were highest among those aged 0–6 months (20.98/1000 person days) and 37–48 months (20.77/1000 person days). This differed from ITFC, where children aged between 25–36 months (23.31/1000 person days) had the highest mortality rate.

**Table 2:** Mortality rates per 1000 person days in IPD and ITFC, AGH, 2016–2018.

		IPD				ITFC			
		Person time of exposure (per 1000 days)	Number of deaths	Mortality rate / 1000 person days	95% CI	Person time of exposure (per 1000 days)	Number of deaths	Mortality rate / 1000 person days	95% CI
**Overall**		53.68	887	16.52	15.47–17.65	61.72	1010	16.36	15.39–17.40
**Age group**	0–6 months	8.30	174	20.98	18.08–24.34	2.53	35	13.83	9.93–19.26
	7–12 months	13.36	201	15.05	13.11–17.28	19.29	259	13.43	11.89–15.17
	13–24 months	16.89	228	13.50	11.86–15.37	31.96	545	17.05	15.67–18.54
	25–36 months	7.65	140	18.29	15.50–21.58	6.43	150	23.31	19.86–27.36
	37–48 months	3.27	68	20.77	16.38–26.34	0.82	11	13.47	7.46–24.34
	49–60 months	2.15	40	18.59	13.63–25.34	0.34	5	14.53	6.05–34.90
	5+ years	2.06	36	17.44	12.58–24.17	0.35	5	14.36	5.98–34.51
**Sex**	Female	24.48	400	16.34	14.82–18.03	28.83	508	17.62	16.16–19.22
	Male	29.21	487	16.76	15.26–18.22	32.90	502	15.26	13.98–16.66
**Year**	2016	11.49	212	18.46	16.13–21.12	16.65	248	14.90	13.15–16.87
	2017	19.98	308	15.41	13.78–17.23	19.35	336	17.36	15.60–19.32
	2018	22.21	367	16.52	14.92–18.30	25.72	426	16.56	15.06–18.21
**Season**	Dry season	38.63	632	16.36	15.13–17.69	43.10	620	14.38	13.30–15.56
	Rainy season	15.06	255	16.94	14.98–19.15	18.62	390	20.94	18.96–23.13
**Time since**	<24	13.31	235	17.66	15.54–20.07	10.58	124	11.72	9.83–13.97
**admission**	24<48	12.22	246	20.14	17.77–22.82	10.17	201	19.77	17.22–22.70
**(hours)**	48<72	8.57	154	17.96	15.34–21.04	9.45	145	15.34	13.04–18.05
	72<96	5.73	87	15.19	12.31–18.75	8.05	122	15.16	12.69–18.10
	≥96	13.86	165	11.91	10.22–13.87	23.47	418	17.81	16.18–19.60
**Diagnosis**	Malaria	27.41	465	16.96	15.49–18.58			n/a	
	Sepsis	2.69	99	36.81	30.23–44.83				
	LRTI	3.96	80	20.19	16.22–25.13				
	Meningitis	1.38	45	32.71	24.42–43.81				
	Measles	7.37	38	5.16	3.75–7.09				
	Neonatal disease	1.37	37	27.10	19.63–37.40				
	Gastroenteritis	3.34	27	8.07	5.54–11.77				
	Tetanus	0.52	18	34.34	21.64–54.51				
	Liver disease	0.09	12	139.21	79.10–245.13				
	Anaemia	0.19	10	52.44	28.21–97.46				
	Other	3.88	52	13.39	10.20–17.57				
**Patient**	Lead-affected villages	4.13	19	4.61	2.94–7.22	1.11	15	13.50	8.14–22.39
**origin**	Other villages	49.56	868	17.52	16.39–18.72	60.60	994	16.40	15.41–17.45

CI–confidence interval; LRTI–lower respiratory tract infection.

The mortality rate in ITFC was higher in female patients compared to male patients (17.6 vs 15.3/1000 person days), although this difference was not significant. In IPD, there was little difference in the observed mortality rates between female and male patients (16.34 vs 16.76/1000 person days).

In IPD, the highest mortality rates were observed in 2016 (18.46/1000 person days), while they were lowest in 2017 (15.41/1000 person days) and increased again in 2018 (16.52/1000 person days). In ITFC, mortality rates were lowest in 2016 (14.90/1000 person days), highest in 2017 (17.36/1000 person days), and decreased in 2018 (16.56/1000 person days) again. There was little observed difference in the mortality rate in IPD between dry and rainy season. However, in ITFC the mortality rate was significantly higher during rainy season (20.94 vs 14.38/1000 person days, p<0.0001).

The mortality rate was highest at 24–48 h of admission for both IPD (20.14/1000 person days) and ITFC (19.77/1000 person days). Mortality rates for admissions lasting ≤24h (17.66 vs 11.72/1000 person days; p = 0.0001) and ≥96h (11.91 vs 17.81/1000 person days; p<0.0001) were significantly higher and lower, respectively, in IPD compared to ITFC.

In IPD, the mortality rate was highest in patients with a primary diagnosis of liver disease (139.21/1000 person days), followed by anaemia (52.44/1000 person days), sepsis (36.81/1000 person days), tetanus (34.34/1000 person days) and meningitis (32.71/1000 person days) with sepsis and meningitis also being among the top 5 morbidities. For the other most common diseases seen in IPD, the mortality rate was 16.96/1000 person days for malaria, 20.2/1000 person days for LRTI, and 5.16/1000 person days for measles.

The mortality rate was lower for patients coming from lead-affected villages compared to other villages for patients seen in both IPD (4.61 vs 17.52/1000 person days) and ITFC (13.50 vs 16.40/1000 person days).

**Fig 2:**
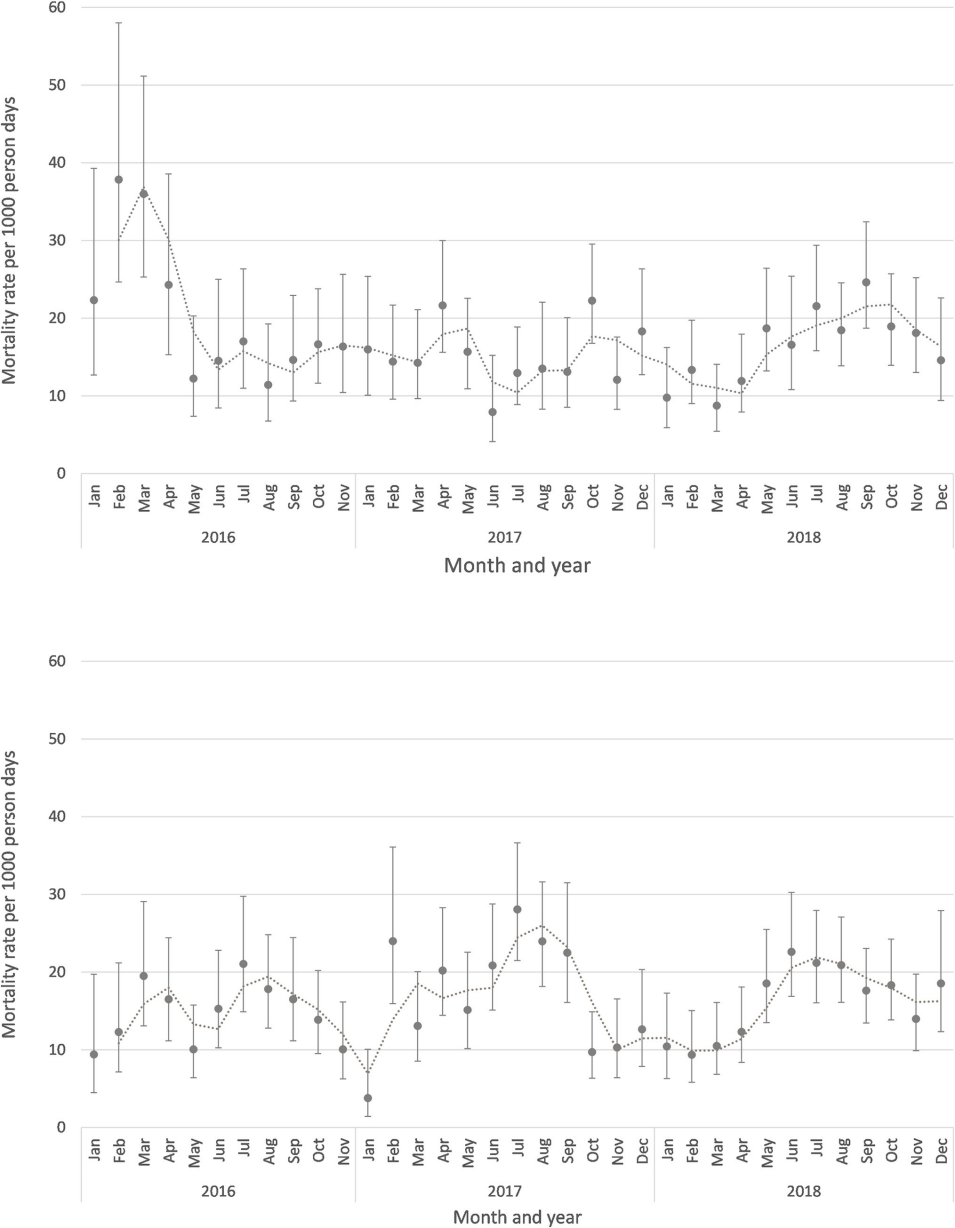
Mortality rates over time in IPD (A) and ITFC (B). Mortality rates per 1000 person days and 95% confidence intervals in IPD (A) and ITFC (B), by month and year of patient exit. Dotted line indicates two months moving average.

The monthly mortality rates per 1000 person days show a seasonal pattern in ITFC with peak rates during rainy season (July, August and September), which was not observed in IPD (Fig 2A and 2B).

### Factors associated with mortality

In the multivariable analysis, the mortality rate in IPD was 15% lower in 2017 and 22% lower in 2018 compared to 2016 (Table 3). The mortality rates were 60%, 87%, 66% and 39% higher within the first 24, 24–48, 48–72 and 72-96h, respectively, compared to ≥96h stay in hospital. The mortality rates were lower with a primary diagnosis of measles (68% lower) and gastroenteritis (51% lower) compared to malaria, whereas a primary diagnosis of LRTI (1.3 times higher), sepsis (2.3 times higher), neonatal disease (1.75 times higher), meningitis (2.3 times higher), anaemia (3.3 times higher) and liver disease (7.8 times higher) increased the mortality rates compared to malaria. Patients coming from lead-affected villages had 70% lower mortality rates compared to those from other villages.

**Table 3:** Univariate and multivariable Poisson regression of mortalities in IPD and ITFC, AGH, 2016–2018.

		IPD						ITFC					
		RR	95% CI	P value	aRR	95% CI	P value	RR	95% CI	P value	aRR	95% CI	P value
**Age groups**	0–6 months	1.20	0.84–1.72	0.0002	1.00	0.69–1.46	0.029	0.96	0.38–2.46	<0.0001	0.95	0.37–2.44	0.0006
	7–12 months	0.86	0.61–1.23		1.00	0.70–1.44		0.93	0.39–2.27		0.91	0.38–2.21	
	13–24 months	0.77	0.54–1.10		0.93	0.65–1.33		1.18	0.49–2.86		1.12	0.46–2.70	
	25–36 months	1.05	0.73–1.51		1.26	0.86–1.83		1.62	0.67–3.96		1.51	0.62–3.67	
	37–48 months	1.19	0.80–1.78		1.42	0.94–2.14		0.94	0.33–2.70		0.89	0.31–2.56	
	49–60 months	1.07	0.68–1.67		1.11	0.71–1.75		1.01	0.29–3.49		0.97	0.28–3.36	
	5+ years	1.00			1.00			1.00			1.00		
**Sex**	Female	0.98	0.86–1.12	0.77	1.00	0.88–1.14	0.98	1.15	1.02–1.31	0.022	1.14	1.01–1.29	0.036
	Male	1.00			1.00			1.00			1.00		
**Year**	2016	1.00		0.13	1.00		0.024	1.00		0.18	1.00		0.11
	2017	0.84	0.70–0.99		0.85	0.71–1.01		1.17	0.99–1.37		1.19	1.01–1.29	
	2018	0.90	0.76–1.06		0.78	0.66–0.93		1.11	0.95–1.30		1.07	0.92–1.25	
**Season**	Dry season	1.00		0.64	1.00		0.43	1.00		<0.0001	1.00		<0.0001
	Rainy season	1.04	0.90–1.20		1.06	0.91–1.24		1.46	1.28–1.65		1.41	1.25–1.61	
**Time since**	<24	1.48	1.22–1.81	<0.0001	1.60	1.31–1.96	<0.0001	0.66	0.54–0.80	<0.0001	0.65	0.53–0.79	<0.0001
**admission**	24<48	1.69	1.39–2.06		1.87	1.53–2.29		1.11	0.94–1.31		1.10	0.93–1.30	
**(hours)**	48<72	1.51	1.21–1.88		1.66	1.33–2.07		0.86	0.71–1.04		0.85	0.70–1.03	
	72<96	1.28	0.98–1.65		1.39	1.07–1.80		0.85	0.70–1.04		0.85	0.69–1.04	
	≥96	1.00			1.00			1.00			1.00		
**Diagnosis**	Sepsis	2.17	1.75–2.70	<0.0001	2.33	1.85–2.94	<0.0001	n/a					
	LRTI	1.19	0.94–1.51		1.30	1.02–1.66							
	Meningitis	1.93	1.42–2.62		2.29	1.66–3.16							
	Measles	0.30	0.22–0.42		0.32	0.23–0.44							
	Neonatal disease	1.60	1.14–2.23		1.75	1.19–2.56							
	Gastroenteritis	0.48	0.32–0.70		0.49	0.33–0.72							
	Tetanus	2.02	1.26–3.24		2.30	1.43–3.72							
	Liver disease	8.21	4.63–14.56		7.77	4.33–13.92							
	Anaemia	3.09	1.65–5.78		3.26	1.74–6.10							
	Other	0.79	0.59–1.05		0.88	0.65–1.17							
	Malaria	1.00			1.00								
**Patient origin**	Lead-affected villages	0.26	0.17–0.41	<0.0001	0.30	0.19–0.48	<0.0001	0.82	0.49–1.40	0.44	0.86	0.52–1.44	0.56
	Other villages	1.00			1.00			1.00			1.00		

RR—unadjusted rate ratio; aRR—adjusted rate ratio; CI—confidence interval; P value from Poisson regression model; Adjusted analyses are adjusted for all variables included in the adjusted Poisson model; LRTI–lower respiratory tract infection.

In ITFC, females had 14% higher mortality rates than males (Table 3). ITFC patients had a 41% higher mortality rate during rainy season compared to dry season. Patients in ITFC had 35% lower mortality rates within the first 24h compared to those at ≥96h.

### LTFU rates

The LTFU rates were highest among younger age groups (Table 4), but IDP and ITFC showed similar observed LTFU rates. LTFU rates between males and females were similar. The highest LTFU rates were observed in 2017 for both wards, the lowest in 2018 for IPD and in 2016 for ITFC. There was little difference in LTFU rates between the dry and the rainy season. LTFU rates were increasing with time of admission in both IPD and ITFC. LTFU rates were highest for patients with a primary diagnosis of neonatal disease, pertussis and poisoning. More patients with a geolocation of origin in one of the lead-affected villages were LTFU.

**Table 4:** LTFU rates per 1000 person days in IPD and ITFC, AGH, 2016–2018.

		IPD				ITFC			
		Person time of exposure (per 1000 days)	Number of patients LTFU	LTFU rate / 1000 person days	95% CI	Person time of exposure (per 1000 days)	Number of patients LTFU	LTFU rate / 1000 person days	95% CI
**Overall**		53.68	373	6.95	6.28–7.69	61.72	399	6.46	5.86–7.13
**Age group**	0–6 months	8.30	76	9.16	7.32–11.47	2.53	19	7.51	4.79–11.77
	7–12 months	13.36	107	8.01	6.63–9.68	19.29	124	6.43	5.39–7.67
	13–24 months	16.89	117	6.93	5.78–8.31	31.96	208	6.51	5.68–7.46
	25–36 months	7.65	34	4.44	3.17–6.22	6.43	42	6.53	4.82–8.83
	37–48 months	3.27	20	6.11	3.94–9.47	0.82	5	6.13	2.55–14.72
	49–60 months	2.15	5	2.32	0.97–5.58	0.34	0	0	n/a
	5+ years	2.06	14	6.78	4.02–11.45	0.35	1	2.87	0.40–20.39
**Sex**	Female	24.48	157	6.41	5.49–7.50	28.83	186	6.45	5.59–7.45
	Male	29.21	216	7.40	6.47–8.45	32.90	213	6.47	5.66–7.41
**Year**	2016	11.49	84	7.31	5.90–9.06	16.65	83	4.99	4.02–6.18
	2017	19.98	162	8.11	6.95–9.46	19.35	139	7.18	6.08–8.48
	2018	22.21	127	5.72	4.80–6.80	25.72	177	6.88	5.94–7.97
**Season**	Dry season	38.63	274	7.09	6.30–7.99	43.10	278	6.45	5.73–7.25
	Rainy season	15.06	99	6.58	5.40–8.01	18.62	121	6.50	5.44–7.76
**Time since**	<24	13.31	34	2.55	1.83–3.58	10.58	19	1.80	1.15–2.81
**admission**	24<48	12.22	49	4.01	3.03–5.31	10.17	34	3.34	2.39–4.68
**(hours)**	48<72	8.57	36	4.20	3.03–5.82	9.45	29	3.07	2.13–4.42
	72<96	5.73	54	9.43	7.22–12.31	8.05	48	5.96	4.49–7.91
	≥96	13.86	200	14.43	12.56–16.58	23.47	269	11.46	10.17–12.91
**Diagnosis**	Malaria	27.41	124	4.52	3.79–5.39	n/a			
	LRTI	3.96	24	6.06	4.06–9.04				
	Sepsis	2.69	22	8.18	5.39–12.42				
	Gastroenteritis	3.34	22	6.58	4.33–9.99				
	Neonatal disease	1.37	16	11.72	7.18–19.13				
	Measles	7.37	15	2.04	1.23–3.38				
	Meningitis	1.38	11	8.00	4.43–14.44				
	Pertussis	0.30	7	23.63	11.27–49.57				
	Skin disease	0.71	6	8.44	3.79–18.78				
	Poisoning	0.12	4	33.28	12.49–88.67				
	Other	3.56	26	7.31	4.98–10.73				
**Patient**	Lead-affected villages	4.13	48	11.64	8.77–15.44	1.11	16	14.40	8.82–23.50
**origin**	Other villages	49.56	325	6.56	5.88–7.31	60.60	383	6.32	5.72–6.99

CI–confidence interval; LRTI–lower respiratory tract infection.

### Factors associated with LTFU

For patients in IPD we observed lower LTFU rates within the first 72h after admission (Table 5). Patients with a primary diagnosis of measles (56% lower) had lower LTFU rates. LTFU rates were higher in patients with a primary diagnosis of pertussis (4.6 times higher) or poisoning (7.1 times higher).

**Table 5:** Univariate and multivariable Poisson regression of patients LTFU in IPD and ITFC, AGH, 2016–2018.

		IPD						ITFC					
		RR	95% CI	P value	aRR	95% CI	P value	RR	95% CI	P value	aRR	95% CI	P value
**Age groups**	0–6 months	1.35	0.76–2.38	0.0004	1.26	0.64–2.45	0.013	2.61	0.35–19.53	0.45	2.59	0.35–19.34	0.40
	7–12 months	1.18	0.68–2.06		1.29	0.67–2.48		2.24	0.31–16.02		2.24	0.31–16.05	
	13–24 months	1.02	0.59–1.78		1.07	0.56–2.05		2.27	0.32–16.16		2.19	0.31–15.66	
	25–36 months	0.66	0.35–1.22		0.75	0.36–1.54		2.27	0.31–16.51		2.18	0.30–15.90	
	37–48 months	0.90	0.46–1.78		0.83	0.36–1.91		2.13	0.25–18.26		1.96	0.23–16.83	
	49–60 months	0.34	0.12–0.95		0.29	0.08–1.04		*			*		
	5+ years	1.00			1.00			1.00			1.00		
**Sex**	Female	0.87	0.71–1.07	0.17	0.83	0.65–1.06	0.13	1.00	0.82–1.21	0.97	1.00	0.82–1.22	0.97
	Male	1.00			1.00			1.00			1.00		
**Year**	2016	1.00		0.011	1.00		0.06	1.00		0.016	1.00		0.004
	2017	1.11	0.85–1.44		0.73	0.53–1.00		1.44	1.10–1.89		1.55	1.18–2.03	
	2018	0.78	0.59–1.03		0.69	0.51–0.95		1.38	1.06–1.79		1.40	1.08–1.82	
**Season**	Dry season	1.00		0.52	1.00		0.43	1.00		0.95	1.00		0.59
	Rainy season	0.93	0.74–1.17		1.11	0.85–1.45		1.01	0.81–1.25		1.06	0.86–1.32	
**Time since**	<24	0.18	0.12–0.25	<0.0001	0.28	0.19–0.41	<0.0001	0.16	0.10–0.25	<0.0001	0.15	0.10–0.24	<0.0001
**admission**	24<48	0.28	0.20–0.38		0.43	0.31–0.61		0.29	0.20–0.42		0.29	0.20–0.41	
**(hours)**	48<72	0.29	0.20–0.41		0.35	0.23–0.54		0.27	0.18–0.39		0.26	0.18–0.39	
	72<96	0.65	0.48–0.88		0.90	0.64–1.26		0.52	0.38–0.71		0.51	0.38–0.70	
	≥96	1.00						1.00					
**Diagnosis**	Sepsis	1.81	1.15–2.85	<0.0001	1.59	0.99–2.57	<0.0001	n/a					
	LRTI	1.34	0.86–2.07		1.11	0.71–1.75							
	Meningitis	1.78	0.95–3.27		1.40	0.74–2.65							
	Measles	0.45	0.26–0.77		0.44	0.26–0.76							
	Neonatal disease	2.59	1.54–4.36		1.75	0.96–3.20							
	Gastroenteritis	1.45	0.92–2.29		1.37	0.86–2.16							
	Pertussis	5.22	2.44–11.19		4.56	2.11–9.87							
	Skin disease	1.86	0.82–4.23		1.37	0.59–3.15							
	Poisoning	7.36	2.72–19.91		7.12	2.48–20.45							
	Other	1.62	1.06–2.47		1.43	0.92–2.23							
	Malaria	1.00			1.00								
**Patient origin**	Lead-affected villages	1.77	1.31–2.40	0.0006	0.90	0.55–1.48	0.68	2.28	1.38–3.76	0.004	2.48	1.50–4.10	0.002
	Other villages	1.00			1.00			1.00					

RR—unadjusted rate ratio; aRR—adjusted rate ratio; CI—confidence interval; P value from Poisson regression model; Adjusted analyses are adjusted for all variables included in the adjusted Poisson model; LRTI–lower respiratory tract infection.

*no events observed.

LTFU rates were higher for patients in ITFC in 2017 (1.6 times higher) and 2018 (1.4 times higher) when compared to 2016 (Table 5). Within the first 96h after admission LTFU rates were lower compared to greater than 96h. Patients in ITFC had 2.5 times higher LTFU rates if they originated from a lead-affected village.

## Discussion

We have shown that mortality rates in AGH ITFC and IPD were in the range of those observed in similar settings, but also consistently high over the study period [3–7, 25]. The observed LTFU rates were below those reported in other countries [6, 16]. The adjusted mortality rate ratio in IPD decreased over time. We think that one of the contributing factors to this might have been the provision of higher quality care, including aspects such as Emergency Triage, Assessment and Treatment (ETAT) training, increased number of staff, Infection Prevention and Control training and Antimicrobial Resistance monitoring for sepsis patients. However, this was not explored in detail and understanding the impact of each of these interventions would require separate evaluation.

Although mortality was highest within the first few days of admission to IPD, this was not the case in ITFC. Whereas LTFU rates in both wards were lowest within the first 24 hours of admission. We showed that in IPD patients, lower mortality was associated with their village of residence being one that had benefitted from a previous lead-poisoning intervention. In the ITFC, we observed an association between the rainy season and mortality and an increase in LTFU rates over time compared to the first year of the study period. ITFC patients also had higher LTFU rates when they lived in a lead-affected village.

Identifying specific predictors of negative patient outcome is challenging. Other studies have indicated that accessibility to, and awareness of available healthcare may influence the outcome of the paediatric patients [11, 12]. Our study, looking at patient characteristics related to accessibility and awareness, shows the impact of these factors on mortality and LTFU in paediatric patients admitted to AGH. The factors we considered in our study included reason for admission, year of admission, time from admission, and village of residence. However, distance travelled to the hospital, which may have an additional impact on clinical presentation and outcome of the patient, has not been assessed in this study.

Higher mortality rates within the first 96h since admission in IPD were similar to the findings of a study in Mali, where more children died within the first three days of admission [5]. Although symptom onset and duration were not recorded in our dataset, based on geolocation and outcome, the higher mortality rate may suggest that these patients may present late to hospital and are thus sicker on arrival. A recent study from Rwanda showed caregivers delayed in seeking healthcare for their children because of the use of traditional healers [26], which might be another aspect of late presentation in the current study, but has not been explored in detail.

The increase in LTFU rates with increasing time of admission in both IPD and ITFC may be due to other obligations of the caretaker requiring them to leave against medical advice. Caretakers usually have other children at home, who may be by themselves, or there might be economic reasons (such as a farm or business) to justify an early departure once the caregiver feels the child is well enough to return home. The reason for higher dropout rates in ITFC could therefore be a result of longer hospital stays for malnutrition [27]. One study from Bangladesh is describing the difference in parental perception of the child’s nutritional status as a main reason to either default or not and it was not possible to identify potential defaulters [28].

The village of residence had a mixed impact on negative outcomes of AGH paediatric patients and this difference may reflect differences in exposure to MSF interventions and the benefits associated with this. Patients from lead-affected villages may benefit from better access to and higher awareness of free healthcare provided by MSF than in other locations, which may reflect the lower mortality rates. This in turn seems to be leading to higher LTFU rates as better access may facilitate an easier return to the hospital in case of deterioration of the patient. In a systematic review on this topic in Sub-Saharan countries it was shown that access to health-care facilities can influence the time between symptom onset and healthcare seeking [29]. Also financial barriers, distance to facilities, lack of knowledge of available services and perceived shortcomings of facilities, such as drug availability, have been described previously and importantly may vary between different countries and regions [30]. Furthermore, overcrowding of facilities may play a role regarding quality of care [31], which is also reflected by the monthly admission rates, especially in ITFC of AGH.

Seasonal factors were also evident, with an association between the rainy season and mortality in ITFC admissions while LTFU rates were independent of season. The influence of seasonality has been described previously for specific diseases, such as for death of malnutrition or malaria during rainy season [4, 32–35]. These reasons as well as reduced access to healthcare due to poorer road conditions or increasing malaria prevalence in this period could apply to this region of Nigeria.

The high LTFU rates for patients with a primary diagnosis of neonatal disease, pertussis and poisoning are difficult to explain. It may be that parents regard these as more serious illnesses and feel that their child is at greater risk of death. There is evidence from Cameroon that even with serious illnesses like cancer, caretakers choose to take their children to traditional healers [36]. This may be the case in AGH with parents choosing to return home to seek alternative treatment or their departure may be prompted by a desire for death to occur at home. Lower LTFU rates in those with a primary diagnosis of measles may be due to the fact that patients are isolated in a separate ward due to the highly contagious nature of the disease and may receive more attentive care. Health seeking behaviour and patient care are both associated with mortality rates and may be contributing factors in our study.

Our study has some limitations. As the outcome of patients following their exit from the hospital was not known, it was not possible to rule out that some patients subsequently died after referral or LTFU. We performed a sensitivity analysis to confirm robustness of results (S3 and S4 Tables), including all referrals and defaulters as potential deaths and comparing the results of the likelihood ratio tests with the results of the full model. The only variables being different in the sensitivity analysis compared to the full model were age group in IPD, and sex and year in ITFC. As for age group and sex, a different proportion of ages and more male patients would be referred than female ones and this might change significance of the result. Regarding the year, many more patients were referred in 2017 and 2018 than in 2016, which we assume may be due to an improvement to the referral system with more patients being referred and hence, fewer deaths among the referrals in contrast to our assumption. We are aware that mortality after discharge is a huge problem in developing countries. However, this was beyond the scope of our study on inpatient mortality and has been described elsewhere [37, 38]. We considered LTFU when there was no formal discharge, referral or death recorded, including patients with missing information on the outcome of their admission, hence, we may have overestimated LTFU in our analysis. It may be another limitation of our analysis that we did not consider LTFU and death as competing risks in the different models. Only the primary diagnosis was available in our dataset of routinely collected data and as a result we cannot rule out any effect of a secondary diagnosis or co-morbidity. The numbers of some diagnoses were too low to allow meaningful conclusions to be drawn. A future extension of this study could be an analysis of co-morbidities and delays in presentation using medical chart reviews.

## Conclusions

Our data contributes clearer understanding of the situation in the paediatric wards in AGH in Nigeria. Access to and awareness of healthcare are well known factors of mortality and also play a role in our study of negative patient outcomes. Despite a large number of patients included in the study, it is challenging to identify specific predictors for outcome. More detailed data is needed for an exploration of the multifaceted nature of mortality and LTFU. Further study in the form of targeted case audits and qualitative studies would help to better understand the role of health-seeking behaviour, and social and traditional factors in the use of formal healthcare in this part of Nigeria. Our study provides insight for other countries with similar contexts to be considered upon undertaking mortality and LTFU studies.

## Supporting information

S1 Checklist STROBE statement—checklist of items that should be included in reports of observational studies. (DOCX)

S1 Table Grouping of diagnoses in IPD and ITFC. Patients admitted to ITFC are all diagnosed with acute malnutrition, even though some other information might have been added, hence this variable was not used for further analysis in ITFC.
(DOCX)

S2 Table Differences in patient outcome (deceased and LTFU) between IPD and ITFC. (DOCX)

S3 Table Comparison of results for deaths in IPD between multivariable Poisson regression and sensitivity analysis. (DOCX)

S4 Table Comparison of results for deaths in ITFC between multivariable Poisson regression and sensitivity analysis. (DOCX)

## List of abbreviations

AGH: Anka General Hospital
aRR: adjusted rate ratio
ATFC: Ambulatory Therapeutic Feeding Centre
CI: Confidence interval
ETAT: Emergency Triage, Assessment and Treatment
IPD: Inpatient Paediatric Department
ITFC: Inpatient Therapeutic Feeding Centre
LGA: local government area
LTFU: lost to follow-up
MOH: Ministry of Health
MSF: Médecins sans Frontières
OCA: Operational Centre Amsterdam
RR: rate ratio
