# Correction: Exploring factors influencing patient mortality and loss to follow-up in two paediatric hospital wards in Zamfara, North-West Nigeria, 2016–2018

**DOI:** 10.1371/journal.pone.0298879

**Published:** 2024-02-09

**Authors:** Anna Maisa, Abdulhakeem Mohammed Lawal, Tarikul Islam, Chijioke Nwankwo, Bukola Oluyide, Adolphe Fotso, Harriet Roggeveen, Saskia van der Kam, Cono Ariti, Karla Bil, Annick Lenglet

There are errors in the Funding and Competing Interests statements. The correct statements are:

**Funding:** Médecins Sans Frontières (MSF) provided support for this study in the form of salaries for AM, AML, TI, BO, AF, HR, SvdK, KB, and AL. The specific roles of these authors are articulated in the ‘author contributions’ section. MSF was involved in the study design, data collection and analysis, decision to publish, and preparation of the manuscript. Every stage of research and dissemination also received approval from the MSF (OCA) Research Committee.

**Competing Interests:** The authors have read the journal’s policy and have the following competing interests to declare: Médecins Sans Frontières (MSF) provided support for this study in the form of salaries for AM, AML, TI, BO, AF, HR, SvdK, KB, and AL. This does not alter our adherence to *PLOS ONE* policies on sharing data and materials. There are no patents, products in development or marketed products associated with this research to declare.
