# Supplementary material for: Exploring factors influencing patient mortality and loss to follow-up in two paediatric hospital wards in Zamfara, North-West Nigeria, 2016–2018
Source: PLoS One. 2021 Dec 31;16(12):e0262073. doi: 10.1371/journal.pone.0262073 (PMC8719718; doi:10.1371/journal.pone.0262073)
Supplement: S1 Checklist — (DOCX) [file pone.0262073.s001.docx]

STROBE Statement—checklist of items that should be included in reports of observational studies

|  | Item No. | Recommendation | Page  No. | Relevant text from manuscript |
| --- | --- | --- | --- | --- |
| **Title and abstract** | 1 | (*a*) Indicate the study’s design with a commonly used term in the title or the abstract | 2 | We conducted a retrospective cohort review study using routinely collected data of all patient admissions to the IPD and ITFC with known hospital exit status between 2016 and 2018. |
|  |  | (*b*) Provide in the abstract an informative and balanced summary of what was done and what was found | 2 | Methods and results section of abstract describe specifics of the study design and findings |
| Introduction | | | |  |
| Background/rationale | 2 | Explain the scientific background and rationale for the investigation being reported | 3-5 | Introduction provides literature review on the topic and what we already know |
| Objectives | 3 | State specific objectives, including any prespecified hypotheses | 5 | Last paragraph of the introduction states the objectives |
| Methods | | | |  |
| Study design | 4 | Present key elements of study design early in the paper | 5 | First section methods describes the study design |
| Setting | 5 | Describe the setting, locations, and relevant dates, including periods of recruitment, exposure, follow-up, and data collection | 5 -7 | Methods section |
| Participants | 6 | (*a*) *Cohort study*—Give the eligibility criteria, and the sources and methods of selection of participants. Describe methods of follow-up  *Case-control study*—Give the eligibility criteria, and the sources and methods of case ascertainment and control selection. Give the rationale for the choice of cases and controls  *Cross-sectional study*—Give the eligibility criteria, and the sources and methods of selection of participants | 5-7 | Methods section |
|  |  | (*b*) *Cohort study*—For matched studies, give matching criteria and number of exposed and unexposed  *Case-control study*—For matched studies, give matching criteria and the number of controls per case |  |  |
| Variables | 7 | Clearly define all outcomes, exposures, predictors, potential confounders, and effect modifiers. Give diagnostic criteria, if applicable | 5-7 | Methods section |
| Data sources/ measurement | 8* | For each variable of interest, give sources of data and details of methods of assessment (measurement). Describe comparability of assessment methods if there is more than one group | 5-7 | Methods section |
| Bias | 9 | Describe any efforts to address potential sources of bias | 6-7 | Methods section |
| Study size | 10 | Explain how the study size was arrived at | 5-7 | Methods section |

Continued on next page

| Quantitative variables | 11 | Explain how quantitative variables were handled in the analyses. If applicable, describe which groupings were chosen and why | 5-7 | Methods section and supplementary material |
| --- | --- | --- | --- | --- |
| Statistical methods | 12 | (*a*) Describe all statistical methods, including those used to control for confounding | 6-7 | Methods section |
|  |  | (*b*) Describe any methods used to examine subgroups and interactions | 6-7 | Methods section |
|  |  | (*c*) Explain how missing data were addressed | 5-7 | Methods section |
|  |  | (*d*) *Cohort study*—If applicable, explain how loss to follow-up was addressed  *Case-control study*—If applicable, explain how matching of cases and controls was addressed  *Cross-sectional study*—If applicable, describe analytical methods taking account of sampling strategy | 5-7 | Methods section |
|  |  | (*e*) Describe any sensitivity analyses | 27 | Discussion and supplementary material |
| Results | | | | |
| Participants | 13* | (a) Report numbers of individuals at each stage of study—eg numbers potentially eligible, examined for eligibility, confirmed eligible, included in the study, completing follow-up, and analysed | 8-10 | Results and Table 1 |
|  |  | (b) Give reasons for non-participation at each stage | NA |  |
|  |  | (c) Consider use of a flow diagram | NA |  |
| Descriptive data | 14* | (a) Give characteristics of study participants (eg demographic, clinical, social) and information on exposures and potential confounders | 8-10 | Results and Table 1 |
|  |  | (b) Indicate number of participants with missing data for each variable of interest | 10 | Table 1 |
|  |  | (c) *Cohort study*—Summarise follow-up time (eg, average and total amount) | 12-14 | Results and Table 2 |
| Outcome data | 15* | *Cohort study*—Report numbers of outcome events or summary measures over time | 10-14 | Results and Table 2 |
|  |  | *Case-control study—*Report numbers in each exposure category, or summary measures of exposure | NA |  |
|  |  | *Cross-sectional study—*Report numbers of outcome events or summary measures | NA |  |
| Main results | 16 | (*a*) Give unadjusted estimates and, if applicable, confounder-adjusted estimates and their precision (eg, 95% confidence interval). Make clear which confounders were adjusted for and why they were included | 14-15, 17-18, 20-21, 23-24 | Tables 2-5 |
|  |  | (*b*) Report category boundaries when continuous variables were categorized | 14-15, 17-18, 20-21, 23-24 | Tables 2-5 |
|  |  | (*c*) If relevant, consider translating estimates of relative risk into absolute risk for a meaningful time period | NA |  |

Continued on next page

| Other analyses | 17 | Report other analyses done—eg analyses of subgroups and interactions, and sensitivity analyses | 27 | Discussion and supplemental information includes the sensitivity analysis |
| --- | --- | --- | --- | --- |
| Discussion | | | | |
| Key results | 18 | Summarise key results with reference to study objectives | 25-28 | Discussion |
| Limitations | 19 | Discuss limitations of the study, taking into account sources of potential bias or imprecision. Discuss both direction and magnitude of any potential bias | 27-28 | Limitations section in the discussion |
| Interpretation | 20 | Give a cautious overall interpretation of results considering objectives, limitations, multiplicity of analyses, results from similar studies, and other relevant evidence | 25-28 | Discussion |
| Generalisability | 21 | Discuss the generalisability (external validity) of the study results | 25-28 | Discussion and conclusion |
| Other information | |  | | |
| Funding | 22 | Give the source of funding and the role of the funders for the present study and, if applicable, for the original study on which the present article is based | 30 | Funding section in the declarations |

*Give information separately for cases and controls in case-control studies and, if applicable, for exposed and unexposed groups in cohort and cross-sectional studies.

**Note:** An Explanation and Elaboration article discusses each checklist item and gives methodological background and published examples of transparent reporting. The STROBE checklist is best used in conjunction with this article (freely available on the Web sites of PLoS Medicine at http://www.plosmedicine.org/, Annals of Internal Medicine at http://www.annals.org/, and Epidemiology at http://www.epidem.com/). Information on the STROBE Initiative is available at www.strobe-statement.org.
