# Supplementary material for: Exploring factors influencing patient mortality and loss to follow-up in two paediatric hospital wards in Zamfara, North-West Nigeria, 2016–2018
Source: PLoS One. 2021 Dec 31;16(12):e0262073. doi: 10.1371/journal.pone.0262073 (PMC8719718; doi:10.1371/journal.pone.0262073)
Supplement: S1 Table — Patients admitted to ITFC are all diagnosed with acute malnutrition, even though some other information might have been added, hence this variable was not used for further analysis in ITFC. (DOCX) [file pone.0262073.s002.docx]

**S1 Table:** Grouping of diagnoses in IPD and ITFC. Patients admitted to ITFC are all diagnosed with acute malnutrition, even though some other information might have been added, hence this variable was not used for further analysis in ITFC.

| Primary diagnosis in IPD includes (n): | |
| --- | --- |
| Malaria | Malaria (7695) |
| Measles | Measles (1725) |
| Gastroenteritis | Chronic (4) and acute (975) gastroenteritis |
| LRTI | LRTI (891), pneumonia (1), respiratory distress (4) |
| Sepsis | Sepsis (629) |
| Neonatal disease | Neonatal disease (245), perinatal asphyxia (15), Hypoxic ischaemic encephalopathy (5), Down Syndrome (2), necrotizing enterocolitis (2), prematurity (2), cerebral palsy (1) |
| Meningitis | Meningitis (203) |
| URTI | URTI (154), Asthma (7) |
| Skin disease | Skin disease (117), necrotizing fasciitis (2), urticarial (1) |
| Tetanus | Tetanus (60) |
| Pertussis | Pertussis (58) |
| Anaemia | Anaemia (55) |
| Kidney disease | Nephrotic syndrome (28), acute kidney failure (24), chronic kidney disease (2), pyelonephritis (1) |
| Sickle cell disease | Sickle cell disease (51) |
| Convulsion | Febrile convulsion (28), convulsion (20), epilepsy (1), neurological convulsion (1) |
| Poisoning | Lead poisoning (20), poisoning (11), intoxication (1), toxic shock syndrome (1) |
| Typhoid fever | Typhoid fever (31) |
| UTI | UTI (27) |
| Fever | Fever (26) |
| Burn | Burn (24) |
| Otitis Media | Acute or chronic otitis media (24) |
| Liver disease | Hepatic failure (8), hepatitis (6), jaundice (5), liver disease (2) |
| Dehydration | Dehydration (17) |
| Acute Abdomen | Acute abdomen (9), intestinal obstruction (8) |
| Heart disease | Heart failure (8), heart disease (3), congenital malformation of heart (3), cardiac arrest (1) |
| Hypoglycemia | Hypoglycemia (11) |
| TB | TB (9) |
| Other | Abdominal pain (2), acute flaccid paralysis (1), acute malnutrition (22), acute mastoiditis (1), allergy (1), arthritis (5), bacterial infection (4), chicken pox (1), chronic disease (2), coagulation defect (1), conjunctivitis (4), constipation (1), disorder of the CNS (1), disseminated intravascular coagulation (1), empyema (2), epistaxis (1), esophagitis (1), failure to thrive (22), foreign body aspiration (1), fracture (1), HFMD (1), head injury (2), hydrocephalus (1), hypersplenism (1), ileus (1), insect/animal bite/sting (4), intraabdominal swelling (1), localized swelling (1), monkey pox (1), multiorgan failure (1), omphalitis (1), orbital cellulitis (1), osteomyelitis (1), pancreatitis (1), parasitic disease (2), peptic ulcer disease (1), peritonitis (1), phimosis (1), pleural effusion (5), pneumothorax (1), pyomyositis (1), rectal prolapse (1), shock (3), tinea (1), transfusion with circulatory overload (1), trauma (2), tropical splenomegaly syndrome (1), ulcer (2), viral infection (1), vulvovaginitis (1) |
| Primary diagnosis in ITFC includes (n): | |
| Acute malnutrition | SAM (10681), MAM (9), Oedema (2), Kwashiorkor (1) |
| Other | Malaria (10), gastroenteritis (6), LRTI (2), sepsis (6), meningitis (1), skin disease (1), anaemia (1), dehydration (1), transfusion with circulatory overload (1) |
