# Supplementary material for: Exploring factors influencing patient mortality and loss to follow-up in two paediatric hospital wards in Zamfara, North-West Nigeria, 2016–2018
Source: PLoS One. 2021 Dec 31;16(12):e0262073. doi: 10.1371/journal.pone.0262073 (PMC8719718; doi:10.1371/journal.pone.0262073)
Supplement: S2 Table — (DOCX) [file pone.0262073.s003.docx]

**S2 Table:** Differences in patient outcome (deceased and LTFU) between IPD and ITFC.

|  |  | **Total admissions** | | **Deceased** | | | | **P value*** | **LTFU** | | | | **P value*** |
| --- | --- | --- | --- | --- | --- | --- | --- | --- | --- | --- | --- | --- | --- |
|  |  | **IPD** | **ITFC** | **IPD** |  | **ITFC** |  |  | **IPD** |  | **ITFC** |  |  |
|  |  | N | n | n | % | n | % |  | n | % | n | % |  |
| **Overall** |  | 13654 | 10744 | 887 | 6.50 | 1010 | 9.40 |  | 373 | 2.73 | 399 | 3.71 |  |
| **Age groups** | 0-6 months | 1836 | 425 | 174 | 9.48 | 35 | 8.24 | <0.0001 | 76 | 4.14 | 19 | 4.47 | <0.0001 |
|  | 7-12 months | 3503 | 3428 | 201 | 5.74 | 259 | 7.56 |  | 107 | 3.05 | 124 | 3.62 |  |
|  | 13-24 months | 4430 | 5529 | 228 | 5.15 | 545 | 9.86 |  | 117 | 2.64 | 208 | 3.76 |  |
|  | 25-36 months | 1982 | 1114 | 140 | 7.06 | 150 | 13.46 |  | 34 | 1.72 | 42 | 3.77 |  |
|  | 37-48 months | 856 | 129 | 68 | 7.94 | 11 | 8.53 |  | 20 | 2.34 | 5 | 3.88 |  |
|  | 49-60 months | 546 | 60 | 40 | 7.33 | 5 | 8.33 |  | 5 | 0.92 | 0 | 0.00 |  |
|  | 5+ years | 501 | 59 | 36 | 7.19 | 5 | 8.47 |  | 14 | 2.79 | 1 | 1.69 |  |
| **Sex** | Female | 6142 | 5055 | 400 | 6.51 | 508 | 10.05 | 0.024 | 157 | 2.56 | 186 | 3.68 | 0.206 |
|  | Male | 7512 | 5689 | 487 | 6.48 | 502 | 8.82 |  | 216 | 2.88 | 213 | 3.74 |  |
| **Year** | 2016 | 3328 | 2807 | 212 | 6.37 | 248 | 8.84 | 0.798 | 84 | 2.52 | 83 | 2.96 | 0.010 |
|  | 2017 | 5195 | 3645 | 308 | 5.93 | 336 | 9.22 |  | 162 | 3.12 | 139 | 3.81 |  |
|  | 2018 | 5131 | 4292 | 367 | 7.15 | 426 | 9.93 |  | 127 | 2.48 | 177 | 4.12 |  |
| **Season** | Dry season | 9827 | 7332 | 632 | 6.43 | 620 | 8.46 | <0.0001 | 274 | 2.79 | 278 | 3.79 | 0.244 |
|  | Rainy season | 3827 | 3412 | 255 | 6.66 | 390 | 11.43 |  | 99 | 2.59 | 121 | 3.55 |  |
| **Length of** | ≤24 | 384 | 180 | 235 | 61.20 | 124 | 68.89 | <0.0001 | 34 | 8.85 | 19 | 10.56 | 0.002 |
| **stay** | 24-48 | 1055 | 396 | 246 | 23.32 | 201 | 50.76 |  | 49 | 4.64 | 34 | 8.59 |  |
| **(hours)** | 48-72 | 3642 | 716 | 154 | 4.23 | 145 | 20.25 |  | 36 | 0.99 | 29 | 4.05 |  |
|  | 72-96 | 2847 | 1404 | 87 | 3.06 | 122 | 8.69 |  | 54 | 1.90 | 48 | 3.42 |  |
|  | ≥96 | 5726 | 8048 | 165 | 2.88 | 418 | 5.19 |  | 200 | 3.49 | 269 | 3.34 |  |
| **Patient** | Lead-affected villages | 1077 | 214 | 19 | 1.76 | 15 | 7.01 | 0.283 | 48 | 4.46 | 16 | 7.48 | <0.0001 |
| **origin** | Other villages | 12577 | 10528 | 868 | 6.90 | 994 | 9.44 |  | 325 | 2.58 | 383 | 3.64 |  |

* P value determined by Pearson’s chi-squared test
