# Supplementary material for: Exploring factors influencing patient mortality and loss to follow-up in two paediatric hospital wards in Zamfara, North-West Nigeria, 2016–2018
Source: PLoS One. 2021 Dec 31;16(12):e0262073. doi: 10.1371/journal.pone.0262073 (PMC8719718; doi:10.1371/journal.pone.0262073)
Supplement: S3 Table — (DOCX) [file pone.0262073.s004.docx]

**S3 Table:** Comparison of results for deaths in IPD between multivariable Poisson regression and sensitivity analysis.

|  |  | **Multivariable analysis** | | | **Sensitivity analysis** | | |
| --- | --- | --- | --- | --- | --- | --- | --- |
|  |  | **aRR** | **95% CI** | **P value** | **aRR** | **95% CI** | **P value** |
| **Age groups** | 0-6 months | 1.00 | 0.69-1.46 | 0.029 | 0.83 | 0.63-1.08 | 0.11 |
|  | 7-12 months | 1.00 | 0.70-1.44 |  | 0.82 | 0.63-1.07 |  |
|  | 13-24 months | 0.93 | 0.65-1.33 |  | 0.75 | 0.58-0.97 |  |
|  | 25-36 months | 1.26 | 0.86-1.83 |  | 0.89 | 0.67-1.17 |  |
|  | 37-48 months | 1.42 | 0.94-2.14 |  | 1.02 | 0.75-1.39 |  |
|  | 49-60 months | 1.11 | 0.71-1.75 |  | 0.78 | 0.55-1.11 |  |
|  | 5+ years | 1.00 |  |  | 1.00 |  |  |
| **Sex** | Female | 1.00 | 0.88-1.14 | 0.98 | 0.98 | 0.88-1.10 | 0.77 |
|  | Male | 1.00 |  |  | 1.00 |  |  |
| **Year** | 2016 | 1.00 |  | 0.024 | 1.00 |  | <0.0001 |
|  | 2017 | 0.85 | 0.71-1.01 |  | 0.78 | 0.67-0.90 |  |
|  | 2018 | 0.78 | 0.66-0.93 |  | 0.74 | 0.64-0.85 |  |
| **Season** | Dry season | 1.00 |  | 0.43 | 1.00 |  | 0.58 |
|  | Rainy season | 1.06 | 0.91-1.24 |  | 1.04 | 0.91-1.17 |  |
| **Time since** | ≤24 | 1.60 | 1.31-1.96 | <0.0001 | 0.88 | 0.75-1.04 | 0.035 |
| **admission** | 24-48 | 1.87 | 1.53-2.29 |  | 1.13 | 0.97-1.32 |  |
| **(hours)** | 48-72 | 1.66 | 1.33-2.07 |  | 1.01 | 0.85-1.20 |  |
|  | 72-96 | 1.39 | 1.07-1.80 |  | 1.09 | 0.90-1.32 |  |
|  | ≥96 | 1.00 |  |  | 1.00 |  |  |
| **Diagnosis** | Sepsis | 2.33 | 1.85-2.94 | <0.0001 | 2.26 | 1.86-2.75 | <0.0001 |
|  | LRTI | 1.30 | 1.02-1.66 |  | 1.29 | 1.05-1.59 |  |
|  | Meningitis | 2.29 | 1.66-3.16 |  | 1.90 | 1.44-2.51 |  |
|  | Measles | 0.32 | 0.23-0.44 |  | 0.34 | 0.26-0.45 |  |
|  | Neonatal disease | 1.75 | 1.19-2.56 |  | 2.16 | 1.62-2.88 |  |
|  | Gastroenteritis | 0.49 | 0.33-0.72 |  | 0.68 | 0.51-0.91 |  |
|  | Tetanus | 2.30 | 1.43-3.72 |  | 1.64 | 1.06-2.55 |  |
|  | Liver disease | 7.77 | 4.33-13.92 |  | 7.76 | 4.75-12.68 |  |
|  | Anaemia | 3.26 | 1.74-6.10 |  | 3.54 | 2.12-5.91 |  |
|  | Other | 0.88 | 0.65-1.17 |  | 1.80 | 1.50-2.16 |  |
|  | Malaria | 1.00 |  |  | 1.00 |  |  |
| **Patient origin** | Lead-affected villages | 0.30 | 0.19-0.48 | <0.0001 | 0.50 | 0.37-0.66 | <0.0001 |
|  | Other villages | 1.00 |  |  | 1.00 |  |  |

aRR - adjusted rate ratio; CI - confidence interval; P value from Poisson regression model; Adjusted analyses are adjusted for all variables included in the adjusted Poisson model; LRTI – lower respiratory tract infection.
