# Supplementary material for: Exploring factors influencing patient mortality and loss to follow-up in two paediatric hospital wards in Zamfara, North-West Nigeria, 2016–2018
Source: PLoS One. 2021 Dec 31;16(12):e0262073. doi: 10.1371/journal.pone.0262073 (PMC8719718; doi:10.1371/journal.pone.0262073)
Supplement: S4 Table — (DOCX) [file pone.0262073.s005.docx]

**S4 Table:** Comparison of results for deaths in ITFC between multivariable Poisson regression and sensitivity analysis.

|  |  | **Multivariable analysis** | | | **Sensitivity analysis** | | |
| --- | --- | --- | --- | --- | --- | --- | --- |
|  |  | **aRR** | **95% CI** | **P value** | **aRR** | **95% CI** | **P value** |
| **Age groups** | 0-6 months | 0.95 | 0.37-2.44 | 0.0006 | 1.19 | 0.84-1.68 | 0.12 |
|  | 7-12 months | 0.91 | 0.38-2.21 |  | 1.30 | 0.94-1.81 |  |
|  | 13-24 months | 1.12 | 0.46-2.70 |  | 1.24 | 0.89-1.72 |  |
|  | 25-36 months | 1.51 | 0.62-3.67 |  | 1.24 | 0.89-1.73 |  |
|  | 37-48 months | 0.89 | 0.31-2.56 |  | 1.07 | 0.73-1.57 |  |
|  | 49-60 months | 0.97 | 0.28-3.36 |  | 1.23 | 0.80-1.89 |  |
|  | 5+ years | 1.00 |  |  | 1.00 |  |  |
| **Sex** | Female | 1.14 | 1.01-1.29 | 0.036 | 1.01 | 0.97-1.06 | 0.57 |
|  | Male | 1.00 |  |  | 1.00 |  |  |
| **Year** | 2016 | 1.00 |  | 0.11 | 1.00 |  | <0.0001 |
|  | 2017 | 1.19 | 1.01-1.29 |  | 4.85 | 4.47-5.25 |  |
|  | 2018 | 1.07 | 0.92-1.25 |  | 3.91 | 3.61-4.23 |  |
| **Season** | Dry season | 1.00 |  | <0.0001 | 1.00 |  | 0.002 |
|  | Rainy season | 1.41 | 1.25-1.61 |  | 1.08 | 1.03-1.13 |  |
| **Time since** | ≤24 | 0.65 | 0.53-0.79 | <0.0001 | 0.06 | 0.05-0.07 | <0.0001 |
| **admission** | 24-48 | 1.10 | 0.93-1.30 |  | 0.13 | 0.12-0.14 |  |
| **(hours)** | 48-72 | 0.85 | 0.70-1.03 |  | 0.22 | 0.21-0.24 |  |
|  | 72-96 | 0.85 | 0.69-1.04 |  | 0.53 | 0.49-0.56 |  |
|  | ≥96 | 1.00 |  |  | 1.00 |  |  |
| **Patient origin** | Lead-affected villages | 0.86 | 0.52-1.44 | 0.56 | 1.22 | 1.04-1.42 | 0.12 |
|  | Other villages | 1.00 |  |  | 1.00 |  |  |

aRR - adjusted rate ratio; CI - confidence interval; P value from Poisson regression model; Adjusted analyses are adjusted for all variables included in the adjusted Poisson model.
